# Cryptococcal meningitis presenting with bilateral complete ophthalmoplegia: a case report

**DOI:** 10.1186/1756-0500-7-328

**Published:** 2014-05-31

**Authors:** Damith S Liyanage, Lakmini PS Pathberiya, Inuka K Gooneratne, Manjula HPC Caldera, Priyankara WS Perera, Ranjani Gamage

**Affiliations:** 1 Institute of Neurology, National Hospital of Sri Lanka, Colombo, Sri Lanka

**Keywords:** Cryptococcal meningitis, Complete ophthalmoplegia, Thalamic lesions

## Abstract

**Background:**

*Cryptococcus neoformans* is saprophytic encapsulated yeast. Infection is acquired by inhalation of the organism and could be asymptomatic or limited to the lungs, specially in the immunocompetent host. Cryptococcal meningitis is a serious opportunistic infection among post transplant recipients. Cranial nerve palsies and ophthalmoplegia are well known complications of this disease, but bilateral complete ophthalmoplegia is a very rare presentation.

**Case Presentation:**

A Sri Lankan young male, who is a post kidney transplant recipient, presented with bilateral complete ophthalmoplegia and subsequently was diagnosed to have cryptococcal meningitis based on Indian ink stain and culture of cerebrospinal fluid (CSF). His magnetic resonance imaging (MRI) showed bilateral multiple nodular lesions in both basal ganglia and thalami. Brainstem imaging was normal.

**Conclusions:**

Cryptococcal meningitis is a serious fungal infection in post transplant patients. It should be suspected in any immunocompromised patient with fever, headache and focal neurological signs. Bilateral thalamic lesions, inflammation and invasion of the cranial nerves and raised intracranial pressure were thought to be possible mechanisms resulting in bilateral complete ophthalmoplegia in this patient.

## Background

*Cryptococcus neoformans* is saprophytic encapsulated yeast with a worldwide distribution in soil contaminated usually with avian excreta, mostly from pigeons [1]. Under the current classification, there are three serotypes pathogenic to humans. They are *C. neoformans grubii*, *C. neoformans gattii* and *C. neoformans neoformans*[2]. Infection is acquired by inhalation of the organism and could be asymptomatic or limited to the lungs, especially in the immunocompetent host. Haematogenous dissemination, especially to the meninges and fatal outcome occurs in immunocompromised patients, particularly lymphoproliferative disorders/malignancy, diabetes mellitus, steroid therapy and infection with human immunodeficiency virus (HIV) [1].

Oculomotor palsies have been reported previously, commonly in the forms of internuclear ophthalmoplegia or as a part of multiple cranial nerve palsies. We describe a post kidney transplant patient with cryptococcal meningitis who presented with bilateral complete ophthalmoplegia and had bilateral thalamic lesions on magnetic resonance imaging (MRI). He was successfully treated with antifungal agents.

### Case Presentation

**Figure 1:**
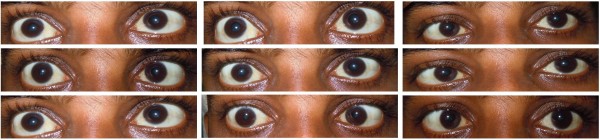
**Eye movements.** Bilateral symmetrical near complete ophthalmoplegia.

A 21-year-old male Sri Lankan patient was transferred from a nephrology unit with a two week history of headache and low grade fever. There was no visual blurring, diplopia, seizures or altered mentation. He had undergone kidney transplant nine months prior to admission. Following that he had been on prednisolone 10 mg daily and mycophenolate mofitil (MMF) 500 mg three times a day.On examination, the patient was alert and febrile. He had neck stiffness. Oculomotor examination revealed bilateral symmetrical near complete ophthalmoplegia with only mild adduction in both eyes possible (Figure 1). Pupils were dilated but reacted to light. There was no ptosis, proptosis or chemosis. Funduscopy revealed engorged veins with preserved disc margin. Visual acuity and language were unaffected. Other cranial nerves were normal. He had no cerebellar signs. His motor and sensory examination was normal.

**Figure 2:**
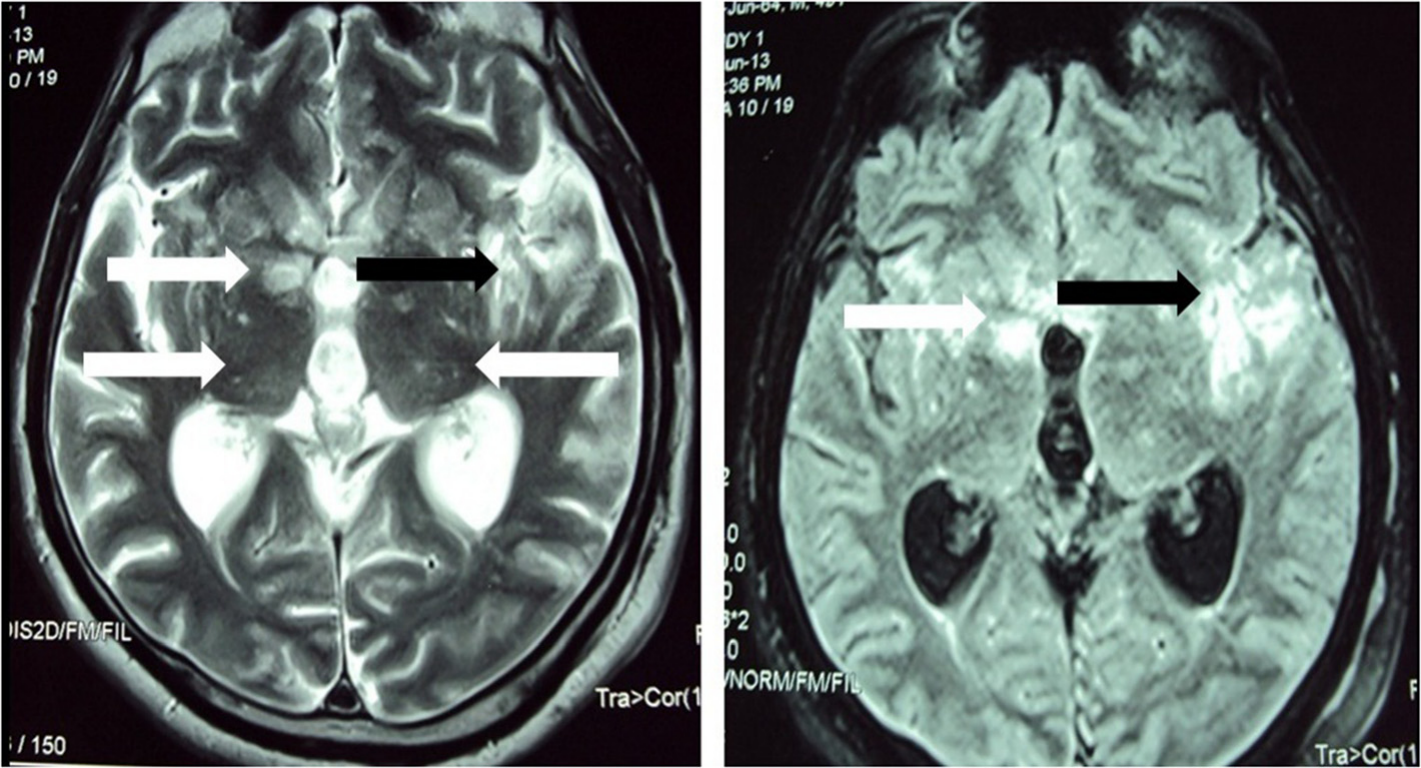
**Fluid attenuated inversion recovery (FLAIR) (Right) and T2-weighted (Left) images of magnetic resonance imaging (MRI) of brain.** Bilateral multiple nodular lesions in basal ganglia and thalamus (white arrows). Ill defined area of signal intensity change in the left temporoparietal region (black arrows).

Initial haematological and biochemical parameters, including complete blood count, inflammatory markers, renal and liver profile were within normal range. Non contrast computed tomography (CT scan) of the brain was normal. Cerebrospinal fluid (CSF) analysis showed high protein levels of 63 mg/dl (ref. range: 15 – 45 mg/dl), lymphocytic pleocytosis (30 lymphocytes/mm^3^ and 5 polymorphonuclearleukocytes/mm^3^) and hypoglycorrhoea (glucose of 1.5 mmol/l in CSF with a plasma value of 5.9 mmol/l). CSF manometry revealed high opening pressure value of 290 mmH_2_O. CSF bacterial culture, smear and culture for acid fast bacilli (AFB) and polymerase chain reaction (PCR) test for tuberculosis (TB) were negative. Indian ink stain of CSF for *Cryptococcus neoformans* was positive and fungal cultures subsequently isolated the same organism. Cryptococcal antigen levels in serum or CSF were not checked due to financial constrains. MRI of the brain demonstrated bilateral multiple nodular lesions in basal ganglia and thalamus. These were hypointense on T1-weighted and hyperintense on fluid attenuated inversion recovery (FLAIR) and T2-weighted images with postcontrast enhancement. Apart from that there was an ill defined area of signal intensity change in the left temporoparietal region with meningeal enhancement. The ventricular system was slightly prominent. No lesions were found in the brainstem (Figure 2).

Intravenous (IV) amphotericin B 0.7 mg/kg/day was started, which was subsequently increased to 1 mg/kg/day. It was continued for six weeks. Initially IV mannitol and IV dexamethasone was given due to raised intracranial pressure, which was tailed off rapidly. By the end of six weeks, CSF pressure had normalized and smears and cultures for *Cryptococcus neoformans* became negative. After six weeks of therapy, he regained full range of eye movements. He was initiated on oral fluconazole 400 mg daily which is to be continued for 10 weeks.

## Discussion

Fungal infections in the organ transplant recipient can cause significant morbidity and possible mortality. The most common cause of CNS infection in the organ transplant population is *C. Neoformans*[3]. The diagnosis of cryptococcal meningitis can be a difficult one as the disease often presents with a sub-acute onset and non specific symptoms and signs. The disease should be suspected in any immunocompromised patient with fever, headache and signs or symptoms referable to the central nervous system (CNS). Our patient presented with fever, headache and bilateral complete ophthalmoplegia. Diagnosis was confirmed by positive CSF cryptococcal indian ink stain and culture and supported by MRI findings.

Cranial neuropathies and ophthalmoplegia are common complications in patients with cryptococcal meningitis, yet bilateral complete ophthalmoplegia is a very rare presentation. Sumit Mohan and colleagues reported a case of cryptococcal meningitis in 2006 with complete loss of vision and hearing as well palsies of the third, sixth and seventh cranial nerves in a sequential manner [4]. Apart from that in our review of the literature we found several references to cases of cryptococcal meningitis with numerous combinations of or isolated second, third, fourth, sixth, seventh and eighth cranial neuropathies. Also there had been reported cases of intranuclear ophthalmoplegia, wall-eyed bilateral internuclear ophthalmoplegia (WEBINO), right Horner's and right hemiparesis syndrome. None of these patients had bilateral complete ophthalmoplegia at presentation or subsequently and there is remarkable paucity of data on bilateral complete ophthalmoplegia in the literature.

MRI of our patient had multiple nodular lesions in basal ganglia and thalamus, but brainstem was spared. These radiological features argue against brainstem pathology as a cause for bilateral complete ophthalmoplegia, which is remarkable. Lesions in basal ganglia and thalamus have been previously reported in cryptococcal meningitis [5,6]. It is believed that the meningeal infection along the base of the skull may involve the adjacent brain parenchyma, giving rise to cryptococcomas or may extend along the Virchow-Robin spaces. The cryptococcal organisms spread through the Virchow-Robin spaces, dilating these spaces, to ultimately propagate in the basal ganglia, internal capsule, thalamus, and brain stem [5,6].

Previous reports have described horizontal and vertical gaze palsies in patients with either unilateral or bilateral thalamic vascular lesions (infarction/haemorrhage) [7,8]. There was no evidence of midbrain involvement in these cases. It has been shown that pathways from the frontal and supplementary eye fields do traverse the medial thalamus. The primate thalamus also has reciprocal inputs to the frontal and supplementary eye fields. This input arises from the internal medullary lamina. The central thalamus is traversed by frontocortical axons, which send collaterals to internal medullary lamina complex neurons. The internal medullary lamina complex also receives afferents from several brain stem populations and the superior colliculus, and it has reciprocal connections with the inferior parietal pole [7]. The possible mechanism of the horizontal gaze disturbances is interruption of the descending fibers from the frontal eye field at the thalamus near the dorsal medial nuclei, internal medullary lamina or medial pulvinar. Vertical gaze dysfunction may result from involvement of the intralaminar and part of the dorsomedial nucleus [8]. Bilateral thalamic involvement may have been a possible mechanism for the ophthalmoplegia without brainstem involvement in our patient. However MRI will not completely exclude coexisting small brainstem lesions.

Cryptococci are thought to physically block the passage of CSF across the arachnoid villi as well as in the subarachnoid spaces. This causes CSF to accumulate causing high intracranial pressure. Increased intracranial pressure leads to compression of the cranial nerves causing neuropathies [4]. Sixth cranial nerve palsy is very common in the setting of raised intracranial pressure as a false localizing sign and it is well reported in cryptococcal meningitis as well [9]. This factor may have contributed to bilateral abductor weakness in our patient. However, ocular palsies seen in this patient solely due to raised intracranial pressure has been poorly described. Inflammation and direct invasion of cranial nerves by the fungus is also thought to be responsible for cranial neuropathies in cryptococcal meningitis [10].

When considering all of these factors, we postulate that bilateral thalamic lesions, inflammation and invasion of the cranial nerves and raised intracranial pressure may have acted individually or in combination to cause bilateral complete ophthalmoplegia in this patient.

Patient’s clinical condition improved with antifungal treatment. Although CSF drainage is recommended as a measure of reducing intracranial pressure in patients with cryptococcal meningitis, it was not done in our patient because CSF pressure was only modestly elevated and as he had clinical improvement with the antifungal agents.

## Conclusions

Cryptococcal meningitis is a serious fungal infection in post transplant patients. It should be suspected in any immunocompromised patient with fever, headache and focal neurological signs. This case illustrates a patient with cryptococcal meningitis with a rare presentation, ie. complete ophthalmoplegia. We have reviewed the literature about previously reported cases of cryptococcal meningitis with cranial neuropathies and ophthalmoplegia. Thalamic involvement, cranial nerve invasion and inflammation and raised intracranial pressure were thought to be responsible for the clinical presentation of our patient. Apart from that this case reiterates the importance of thalamic lesions in producing vertical and horizontal gaze palsies.

## Consent

Written informed consent was obtained from the patient for publication of this Case Report and any accompanying images. A copy of the written consent is available for review by the Editor-in-Chief of this journal.

## Competing interests

The authors declare that they have no competing interests.

## Author’s contributions

DSL, IKG and RG were involved with the diagnosis and the management of the patient. PLSP, WSPP and HPMCC were involved with the literature review. DSL and IKG wrote the manuscript with contributions from all other authors. All authors read and approved the final version of the manuscript.
